# Disseminated Tuberculosis: A Case of Multiple Spread Mass

**DOI:** 10.7759/cureus.11149

**Published:** 2020-10-25

**Authors:** Mariana Formigo, Magda Costa, João Martins, Helena Sarmento, Jorge Cotter

**Affiliations:** 1 Internal Medicine, Hospital Senhora da Oliveira Guimarães, Guimarães, PRT; 2 Oncology, Hospital Senhora da Oliveira Guimarães, Guimarães, PRT

**Keywords:** disseminated tuberculosis, pott disease, antituberculous therapy, tuberculosis

## Abstract

Disseminated tuberculosis is associated with significant morbidity and mortality. It results from a lymphohematogenous dissemination of mycobacterium tuberculosis (MT) and its atypical clinical presentation often delays the diagnosis. Diagnosis is established by identifying MT obtained from a biopsy sample in culture or acid-fast smear. Evidence suggests an initial two-month phase of four-drug therapy followed by a two-drug phase for six to nine months.

A 61-year-old man presented with back lumbar pain. He presented two masses, a left parasternal and a left axillary masses with approximately 6 cm each. He referred a 21% weight loss, anorexia and asthenia. His computed tomography revealed recent lumbar fractures and a left paravertebral space-occupying lesion; hilum and upper lobe masses; inflammatory/infectious micronodules; mediastinal adenomegaly, hypodense lesions in the spleen, sternum and left scapula. Magnetic resonance imaging revealed lumbar vertebral fractures, an anterior epidural collection, left iliac psoas muscle liquid collection. A mass puncture and biopsy were performed, resulting in a positive detection of MT in nucleic acid amplification (NAA). The patient started on quaternary antibacillary therapy with isoniazid, rifampin, pyrazinamide and ethambutol. Bronchofibroscopy revealed an hypervascularized and infiltrated submucosa. Later, histopathology was compatible with chronic granulomatous inflammatory process and bronchial lavage molecular test was positive for MT.

At the moment, he is under two-drug antibacillary therapy with isoniazid and rifampin and masses are regressing.

## Introduction

Disseminated tuberculosis (TB) usually occurs in elderly and immunocompromised subjects and it is associated with significant morbidity and mortality [1]. It may occur in both progressive primary infection or as a reactivation of a latent focus through hematogenous dissemination of mycobacterium tuberculosis (MT) [2].

Miliary TB is characterized by non-specific symptoms and signs as fever, anorexia, night sweats, weight loss, tachypnea, rales and altered mental status [1]. Acute-phase reactants as erythrocyte sedimentation rate and C-reactive protein are usually elevated in patients with miliary TB [1].

TB can involve any organ or tissue in the body. All patients should undergo pulmonary disease evaluation, including a chest radiography, followed by computed tomography (CT) scan, sputum for acid-fast smear and culture, nucleic acid amplification (NAA) testing and tuberculin skin test or Interferon Gamma Release Assay (IGRA). If the results are inconclusive, evaluation via bronchoscopic sampling (including bronchial brushings and/or transbronchial biopsy) is warranted [3]. Patients with extrapulmonary localizing symptoms should undergo directed evaluation on the involved organ system, and radiographic imaging and tissue biopsy may be required to establish a definitive diagnosis.

When facing tuberculous spondylitis (Pott disease), radiographic abnormalities are usually first observed in the anterior aspect of a vertebral body, the opposing vertebra becomes involved and, in some cases, a paravertebral abscess may be seen. Involvement of contiguous vertebrae is common, although it is uncommon to see noncontiguous spinal TB at multiple levels [4]. CT, myelography, and magnetic resonance imaging (MRI) are all useful tools in the diagnosis of musculoskeletal TB [4-7].

Diagnosis is established by identifying MT obtained from a biopsy sample in culture or acid-fast smear [3]. Histopathology of tissue biopsy specimens usually show granulomatous inflammation with granulomas containing epithelioid macrophages, Langhans giant cells and lymphocytes; a characteristic caseation in the center; and organisms may and may not be seen with acid-fast staining [3]. Histopathologic caseating granuloma associated with suggestive clinical and epidemiologic circumstances strongly supports a diagnosis of TB, but it’s not pathognomonic; culture is required to establish a laboratory diagnosis. NAA assays are used to amplify MT RNA or DNA quantity and are sensitive for MT rapid detection in a variety of specimen as blood, sputum, or urine [3]. However, a negative NAA test result should not be used to exclude TB due to commonly false-negative results [3].

Increasing evidence on chemotherapy for extrapulmonary tuberculosis recommend an initial two-month phase of four-drug therapy with isoniazid, rifampin, pyrazinamide and ethambutol (HRZE), followed by a second two-drug phase with isoniazid, rifampin (HR) for six to nine months, depending on clinical response and on each mycobacteria particular drug sensitivity [8,9].

## Case presentation

A 61-year-old man presented at the emergency room with back lumbar pain, he referred having fallen down one month before. His medical background included controlled type 2 diabetes, dyslipidemia and a past history of smoking (about 20 cigarettes/day during 45 years). At the moment he is retired and has previously worked as a bricklayer.

**Figure 1:**
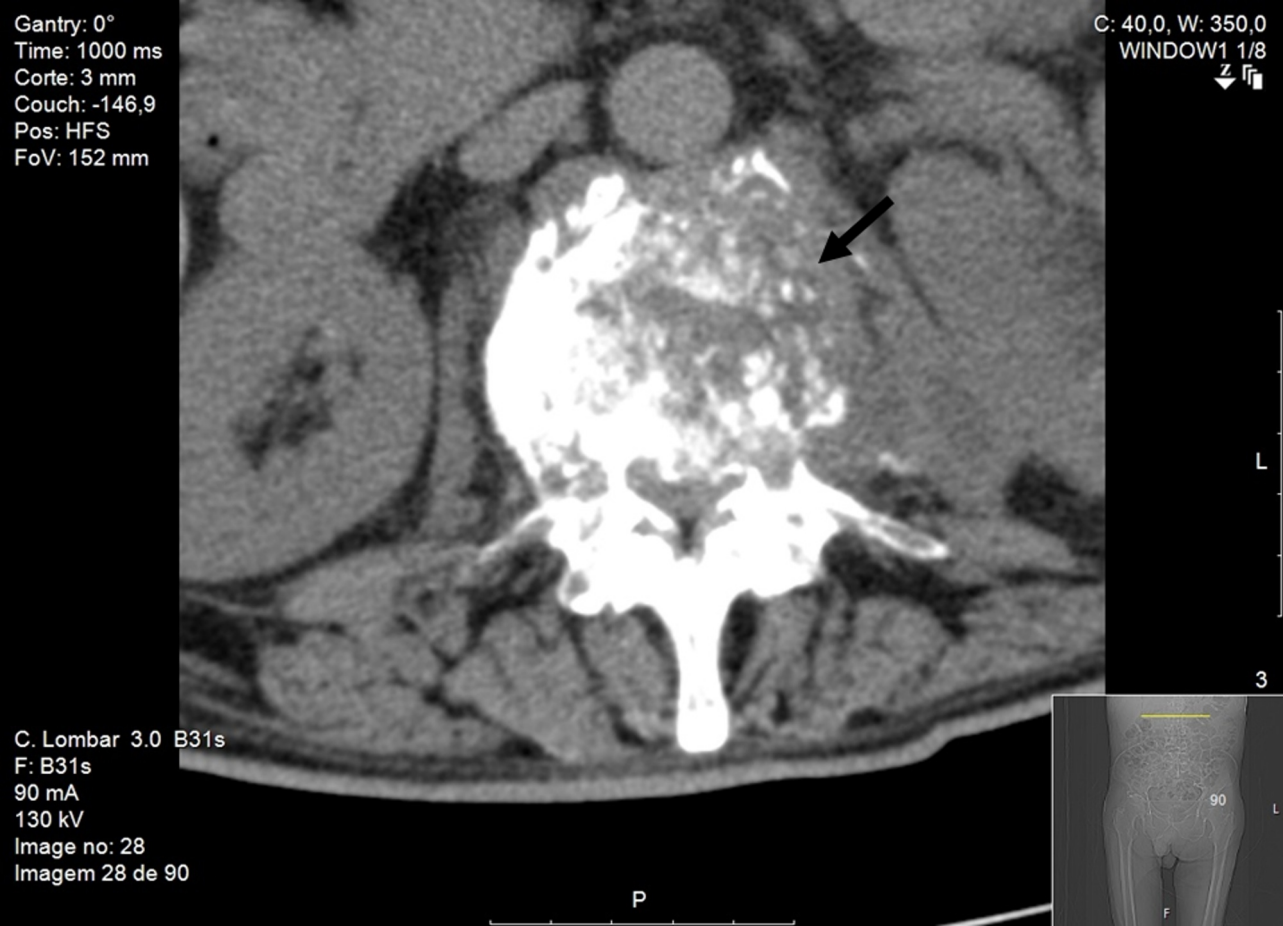
Lumbar mass

He was observed by orthopedic, presenting no motor or neurologic deficits. A lumbar CT revealed L1- L4 recent fractures and a left paravertebral (L1-L3) space-occupying lesion, possibly tumoral etiology (Figure 1).

**Figure 2:**
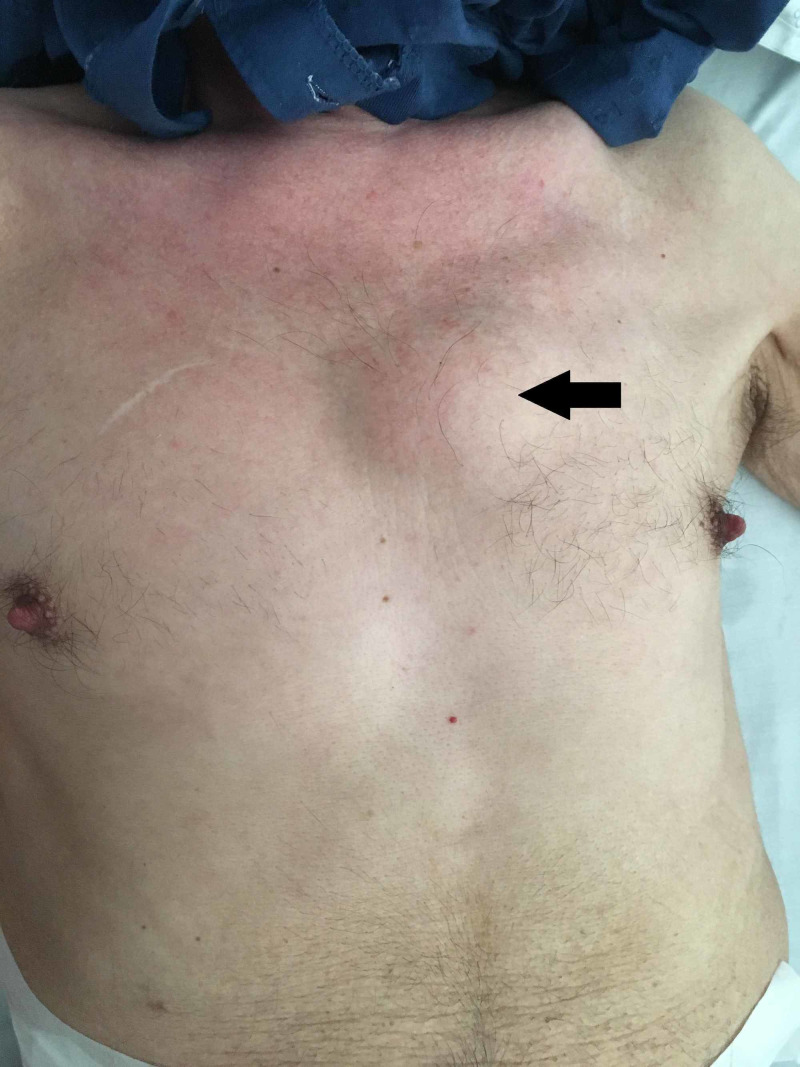
Parasternal mass

**Figure 3:**
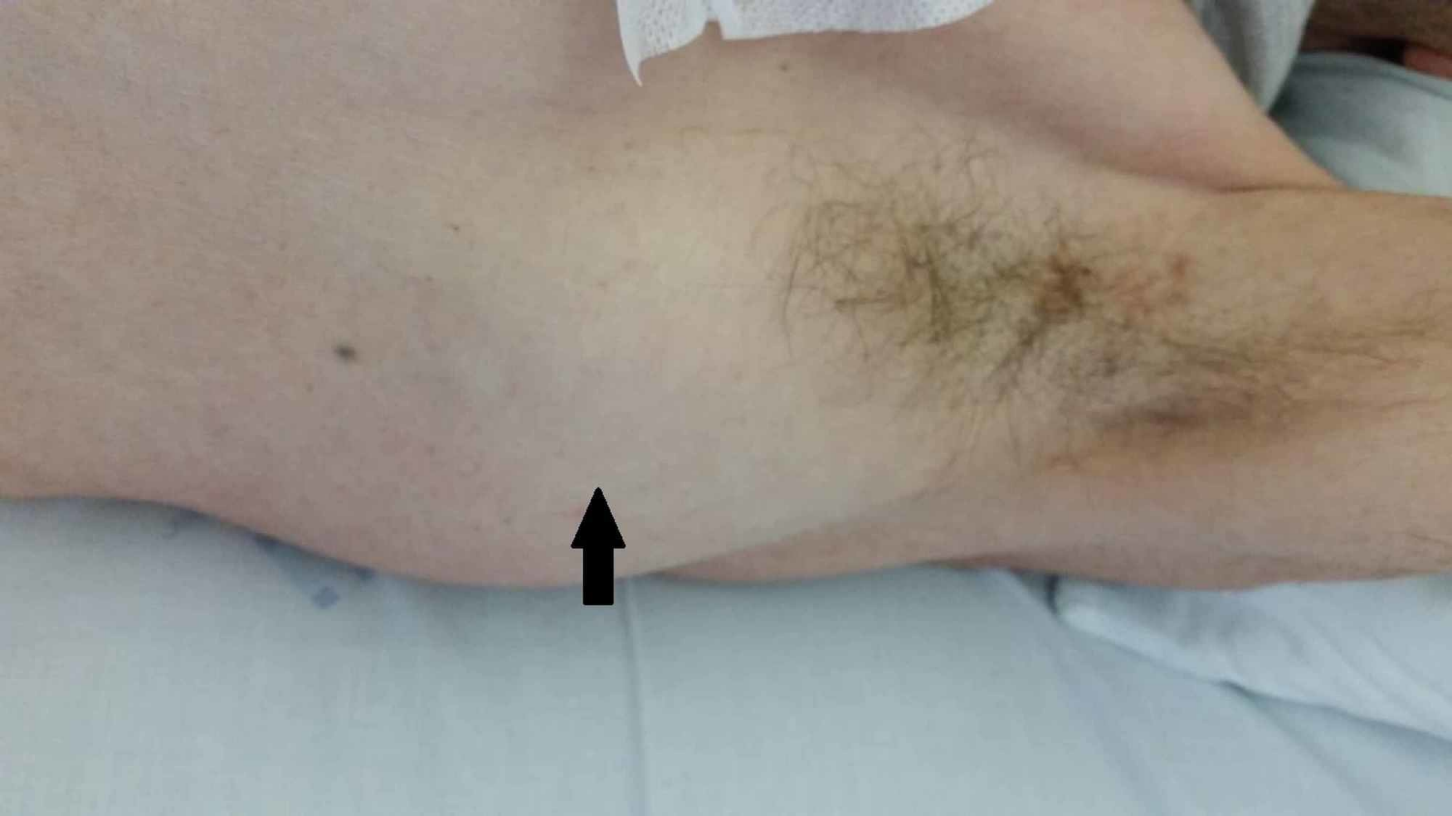
Axillary mass

On that same day, the patient is referred to Internal Medicine. He presented soft, immobile, non-painful, floating left parasternal and left axillary masses with approximately 6 cm (Figures 2, 3).

He also referred a 21% weight loss (74 to 55 Kg in two months) and long-term anorexia and asthenia.

**Figure 4:**
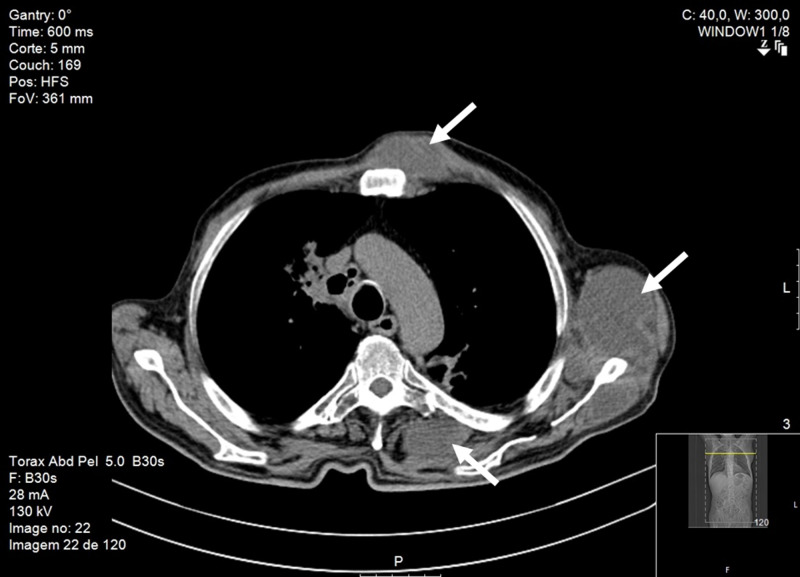
CT scan revealing parasternal, axillary and dorsal masses

A thoracic mass ultrasound revealed a liquefied hypoechoic, heterogeneous, nodular lesion with 6mm of greater diameter, contacting the intercostal space. A thoracoabdominopelvic CT scan revealed one mass in the right hilum and another mass (34 mm) in the left upper lobe (35 mm); inflammatory/infectious micronodules in the left lung; mediastinal adenomegaly, hypodense lesions (largest 41 mm) in the spleen, hypodense lesions on the sternum and left scapula (Figure 4).

Blood analyses revealed an erythrocyte sedimentation rate of 31 mm and a C-reactive protein of 21.4 mg/L. The patient was admitted for study.

**Figure 5:**
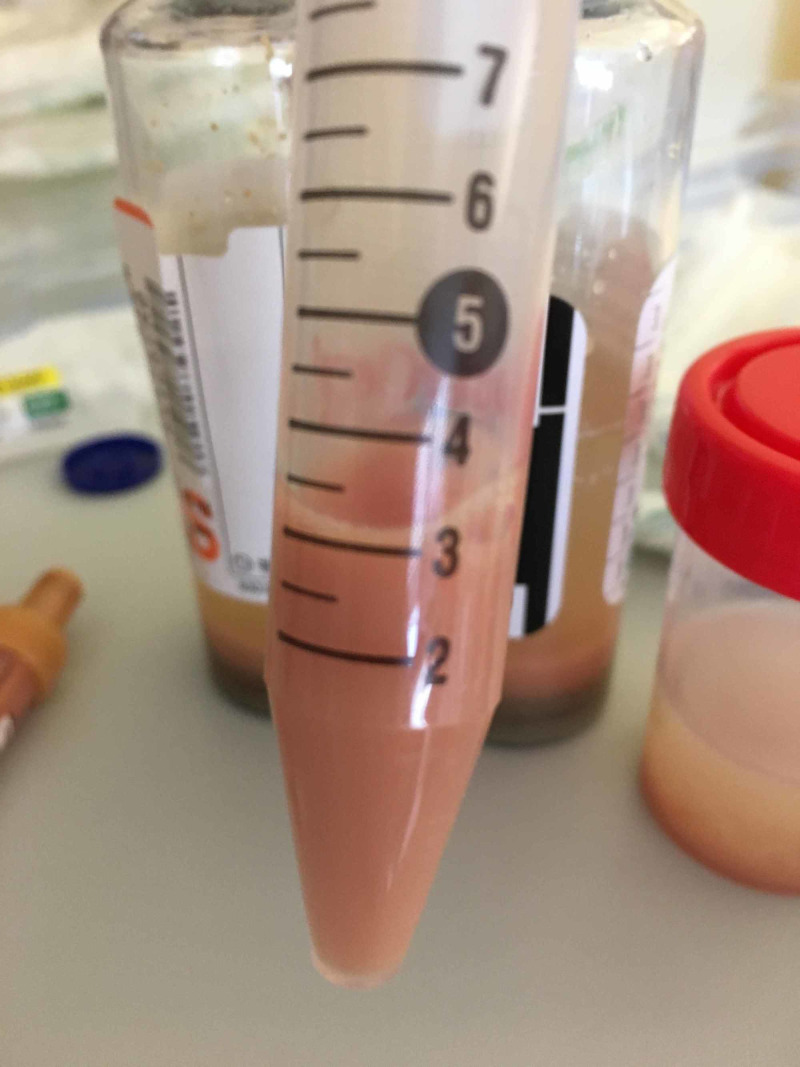
Purulent liquid aspirated from the axillary mass

On the sixth day after admission, a mass punction and biopsy were performed and aspirated 30 cc of a purulent liquid that was sent to microbiology, cytology and histology examinations (Figure 5).

Hepatitis and HIV serological tests were negative. Toxoplasmosis, rubella, cytomegalovirus, herpes simplex and Epstein-Barr virus serologies were negative. He performed a lumbar MRI revealing L1, L2, L3 and L4 vertebral bodies fractures; an anterior epidural collection (D12-L1 and L1-L2) with contrast enhancement; and a liquid collection in the left iliac psoas muscle with peripheral contrast enhancement. He performed a bronchofibroscopy revealing an hypervascularized and infiltrated trachea submucosa extending to carina, left and right primary bronchi; bronchial biopsies were performed at this level and bronchial lavage collected. Microbiologic blood, urine, sputum and pus culture results were negative. IGRA was negative. A transthoracic echocardiogram was normal.

On the 12th day, pus molecular test was positive for MT complex and the patient started on quaternary antibacillary therapy with HRZE. On the 24th day after admission, mass histopathology revealed a predominantly mononuclear polymorphic inflammatory infiltrate and multinucleated giant cells, compatible with chronic granulomatous inflammatory process. Organisms were not seen with acid-fast staining.

During his hospital stay, he was observed by physiatrists and orthopedists, starting rehabilitation using Jewett vest.

He was discharged on the 25th day, forwarded to Internal Medicine, Physiatry and Orthopedic consultations, rehabilitation physiotherapy and Tuberculosis Differentiated Ambulatory Unit.

Later, bronchial lavage molecular test was positive for MT complex.

At the moment, the patient has completed the two-month period of quaternary therapy and is under two-drug antibacillary therapy with HR. Peripheric masses have disappeared completely and lumbar CT reevaluation showed a decrease in mass size.

## Discussion

Disseminated TB results from a lymphohematogenous dissemination of MT and its atypical clinical presentation often delays the diagnosis [2,10]. A high index of suspicion and diagnostic persistence are required for diagnosis [1]. The diagnosis is usually confirmed by MT isolation in sputum, body fluids or biopsy specimens, NAA molecular tests and cytohistopathological examination of tissue biopsy specimens [2,10]. NAA testing should be performed on specimens collected from sites of suspected extrapulmonary TB due to its specificity >95% [3]. Antibacillary susceptibility tests must be carried out for feasible results [2].

Although there is a good response to first-line antibacillary therapy, more studies are necessary to clarify the optimum duration of treatment and the unclear role of adjunctive corticosteroid therapy [8,10].

Prompt TB diagnosis and antibacillary treatment is crucial owing to its associated significant morbidity and mortality [2,4]. In skeletal TB, prompt therapy is important to prevent severe bone and joint destruction or neurologic sequelae when there is spinal involvement [4].

## Conclusions

TB has been well known for centuries, although it is still prevalent in developing and well-developed countries nowadays and it can present unusual presentations. Its natural course can lead to great morbidity and death, so it is important to establish prompt diagnosis and treatment to improve its prognosis.
